# Supplementary material for: Pathogenetic Mechanisms Underlying Major Adverse Cardiac Events in Personality Type D Patients after Percutaneous Coronary Intervention: The Roles of Cognitive Appraisal and Coping Strategies
Source: Diagnostics (Basel). 2023 Nov 2;13(21):3374. doi: 10.3390/diagnostics13213374 (PMC10648350; doi:10.3390/diagnostics13213374)
Supplement: Supplementary file 1 [file diagnostics-13-03374-s001.zip › diagnostics-2633948-supplementary.pdf]

Supplement Table S1.

Baseline characteristics of patients excluded and included 1 year after PCI

Variables		Excluded (n = 21)	Included (n = 91)	p
Male (n, %)		17(80.95)	51(56.1)	0.04
Age, years		63.0[52.0;64.0]	65.0[59.0;70.0]	0.039
BMI, kg/m2		25.6[23.6;26.9]	25.3[21.3;27.7]	0.754
Disability (n, %)		7(33.3)	27(29.7)	0.639
Working (n, %)		8(38.1)	35(38.5)	0.926
Current smoking (n, %)		12(57.1)	38(41.7)	0.161
Smoking experience, years		26.0[11.0;40.0]	20.0[10.0;38.0]	0.199
Hypertension, n (%)		18(85.7)	76(83.5)	0.237
Diabetes mellitus, n (%)		3(14.3)	22(24.2)	0.414
Stroke (n, %)		2(9.5)	8(8.8)	0.766
Myocardial infarction (n, %)		10(47.6)	52(57.1)	0.681
Previous CABG (n, %)		2(9.5)	8(8.8)	0.719
Carotid endarterectomy (n, %)		1(4.8)	1(1.1)	0.221
Angina class	0 (n, %)	2(9.5)	16(17.6)	0.439
	I (n, %)	2(9.5)	9(9.9)	0.944
	II (n, %)	13(61.9)	62(68.1)	0.968
	III (n, %)	2(9.5)	3(3.3)	0.173
Heart failure class NYHA	0 (n, %)	0	7(7.7)	0.209
	I (n, %)	15(71.4)	44(48.4)	0.02
	II (n, %)	4(19.1)	37(40.6)	0.101
	III (n, %)	0	1(1.1)	0.644
**Laboratory indicators**				
Total cholesterol, mmol/l		4.2[3.5;5.6]	4.0[3.5;5.0]	0.441
HDL cholesterol, mmol/l		1.01[0.93;1.12]	1.05[0.85;1.4]	0.802
LDL cholesterol, mmol/l		2.4[2.1;2.6]	2.7[1.8;3.2]	0.504
Triglyceride, mmol/l		1.3[1.2;1.4]	1.3[1.1;1.6]	0.759
Creatinine, µmol/l		87.0[68.0;95.0]	90.5[78.5;111.5]	0.158
Glucose, mmol/l		5.6[5.3;6.5]	6.0[5.3;7.2]	0.427
**Coronarography**				
1-coronary artery disease, n (%)		11(52.4)	66(72.5)	0.072
2-coronary artery disease, n (%)		8(38.1)	18(19.8)	0.073
3-coronary artery disease, n (%)		2(9.5)	7(7.7)	0.781

Data are presented as the median and interquartile range (25 th -75 th percentile) or number [%], as indicated.

Notes: BMI - Body Mass Index, NYHA - New York Heart Association, CABG - coronary artery bypass grafting, HDL - high-density lipoproteins, LDL - low-density lipoproteins.

Supplement Table S2.

Comparative analysis of the initial data of the questionnaire of patients excluded and included 1 year after PCI

Variables	Excluded (n = 21)	Included (n = 91)	p
**DS-14**			
negative affectivity, points	8.67±4.3	10.7±4.6	0.11
social inhibition, points	9.29±4.2	10.1±3.8	0.24
**HADS**			
Personal anxiety, points	4.43±3.1	6.11±3.1	0.021
Depression level, points	3.9±2.8	4.9±3.6	0.358
**Cognitive appraisal (WCQ)**			
Confrontation, points	11.1±4.3	10.1±3.6	0.167
Distancing, points	11.3±3.7	10.9±3.5	0.701
Self-control, points	13.3±4.6	12.8±3.7	0.297
Search for social support, points	13.2±4.4	11.9±3.9	0.102
Taking responsibility, points	9.2±2.9	8.5±2.6	0.111
Avoidance, points	13.9±5.7	12.3±4.01	0.271
Problem solving planning, points	13.7±3.9	13.4±3.2	0.373
Secondary appraisal, points	15.4±5.1	13.1±3.9	0.01

Note: M (SD) = mean (standard deviation).

Supplement Table S3.

Comparative analysis of the questionnaire data "Cognitive assessment of a difficult life situation" in groups of patients with excluded/included

Variables	Excluded (n = 21)	Included (n = 91)	p
Common signs of difficult life situations, points	16.7±5.4	15.6±5.0	0.426
Lack of control of the situation, points	12.3±4.2	10.6±4.6	0.068
Incomprehensibility of the situation, points	13.1±8.4	15.4±6.9	0.383
The need for a quick, active response, points	16.6±3.6	16.0±4.8	0.830
Difficulties in making a decision, points	13.3±4.5	14.3±4.9	0.206
Difficulties in predicting the situation, points	9.3±4.1	10.1±4.3	0.391
Strong emotions, points	14.3±5.5	13.5±5.2	0.821
Future perspective, points	12.8±3.9	12.5±3.7	0.711

Supplement Table S4.

Comparative analysis of the questionnaire data "Types of orientations in difficult situations" in groups of patients with excluded/included

Variables	Excluded (n = 21)	Included (n = 91)	p
Striving for difficulties, points	25.2±5.7	22.5±5.6	0.05
Orientation to high labor intensity, points	11.3±3.5	11.9±2.5	0.261
Targeting threat signals, points	12.5±3.1	12.1±2.9	0.553
Focus on opportunities, points	19.4±3.7	18.2±3.5	0.151
Orientation to obstacles, points	11.9±4.1	12.2±2.9	0.811
Loss orientation, points	15.7±4.4	16.1±5.1	0.939
Resource conservation orientation, points	15.3±2.7	14.8±4.1	0.912
Focus on ignoring difficulties, points	12.9±3.5	11.5±3.6	0.467
